# *Saccharomyces cerevisiae* single-copy plasmids for auxotrophy compensation, multiple marker selection, and for designing metabolically cooperating communities

**DOI:** 10.12688/f1000research.9606.1

**Published:** 2016-09-20

**Authors:** Michael Mülleder, Kate Campbell, Olga Matsarskaia, Florian Eckerstorfer, Markus Ralser

**Affiliations:** 1 Department of Biochemistry and Cambridge Systems Biology Centre, University of Cambridge, Cambridge, UK; 2 Mill Hill Laboratory, The Francis Crick Institute, London, UK; 3 Chalmers University of Technology, Gothenburg, Sweden

**Keywords:** Saccharomyces cerevisiae, centromeric plasmid, auxotrophic markers, self-establishing metabolically cooperating (SeMeCo) communities, metabolism

## Abstract

Auxotrophic markers are useful tools in cloning and genome editing, enable a large spectrum of genetic techniques, as well as facilitate the study of metabolite exchange interactions in microbial communities. If unused background auxotrophies are left uncomplemented however, yeast cells need to be grown in nutrient supplemented or rich growth media compositions, which precludes the analysis of biosynthetic metabolism, and which leads to a profound impact on physiology and gene expression. Here we present a series of 23 centromeric plasmids designed to restore prototrophy in typical *Saccharomyces cerevisiae* laboratory strains. The 23 single-copy plasmids complement for deficiencies in *HIS3, LEU2, URA3, MET17 or LYS2* genes and in their combinations, to match the auxotrophic background of the popular functional-genomic yeast libraries that are based on the S288c strain. The plasmids are further suitable for designing self-establishing metabolically cooperating (SeMeCo) communities, and possess a uniform multiple cloning site to exploit multiple parallel selection markers in protein expression experiments.

## Introduction

Auxotrophic markers are single gene perturbations of essential metabolic pathways, that are exploited in the efficient selection of strains, plasmids and genome editing. Further, they are used in a diverse spectrum of genetic technologies, as their selection is efficient, their use economic, and in contrast to antibiotic selection markers, they do not revert by mutation ^1– 3^. In budding yeast, auxotrophic marker alleles important for histidine, leucine, uracil, methionine, lysine, adenine and tryptophan metabolism have been crossed or cloned into the popular *S. cerevisiae* laboratory strains. Harbouring 5 auxotrophic marker mutations, *his3*Δ1, *leu2*Δ0, *ura3*Δ0 *, lys2*Δ0 or *met17*Δ0 ^4– 9^, strains derived from the S288c background served as the parents of the yeast gene-deletion collection ^5^, and subsequent genetic libraries that are based on this principle. These libraries include gene deletion mutants ^5, 10, 11^, genetically introduced GFP, GST, and TAP fusions ^12– 14^, transposon insertion mutants ^15^, decreased abundance by mRNA perturbation (DAmP) mutants ^16^, Tet-promoter controlled expression ^17^ and the ts-alleles for essential genes ^18, 19^. Furthermore, systematic strain collections of other fungal species including *Schizosaccharomyces pombe* ^20, 21^ and the pathogens *Candida glabrata* ^22^, *Candida albicans* ^23^ or *Neurospora crassa* ^24^, all involve use of auxotrophic markers as well. As a result, auxotrophic backgrounds are omnipresent in a large number of functional genomic experiments, and have been used in a countless number of small-scale experiments, resulting in their ubiquity across yeast molecular biology literature.

In order for a metabolic gene to function as an auxotrophic marker, it needs to be part of a metabolic pathway for which the cells possess an extracellular uptake and a sensing mechanism for the product of the interrupted pathway. Auxotrophic marker mutations are hence associated with metabolites that are readily taken up from the environment. This includes the metabolites exchanged between cells in communal growth, in particular amino acids ^25^. The biosynthesis of amino acids accounts for up to half of the metabolic flux towards biomass, with amino acids making up to 2/3rds of the total mass of polar metabolites ^26, 27^. As a consequence, a shift from self-synthesis to uptake, as enforced by auxotrophy, is not without biological consequence. In fact, most of the genome-wide gene expression is sensitive to epistatic interaction within the *Saccharomyces* metabolic-genetic background ^28^. The physiological effects arising from auxotrophy and complemented marker genes have been highlighted by several yeast labs for more than a decade ^2, 29– 33^. Most importantly, to grow auxotrophic strains, amino acids and nucleotides need to be added to the growth medium. Nutrient supplementation affects not only the interrupted pathway itself, but the biosynthesis of other essential compounds, in particular the enzymatic cofactors, due to the metabolic network responding to perturbation at the systems levels, and hence, affecting multiple metabolic pathways in parallel ^2, 34, 35^. Cell growth has consistently shown to be affected by nutrient supplementation, reflecting the variation in energy costs between biosynthesis and uptake/incorporation of the provided nutrients ^2, 34^. In batch cultures, supplements are also consumed at different rates ^33^. As a consequence, nutrient availability changes during batch culture growth, rendering cells physiologically different between growth phases. In classic molecular biology, the use of a matched auxotrophic background as a wild-type control has been considered sufficient to account for the effects of auxotrophy ^2^. Transcriptomic, proteomic and metabolomic analysis of complemented auxotrophs show however that this is not the case; the metabolic background deficiencies interact epistatically with the majority of the coding genome and in a context dependent manner. The biological explanation for this phenomenon is that metabolism is intrinsically intertwined with the gene expression machinery and is dependent on metabolic flux distribution. The same gene deletion introduced in a different auxotrophic background can hence cause an entirely different transcriptional response, so that a matched parent background is not able to compensate for these effects ^28^.

We here present a series of single copy plasmids derived from the pHLUM minichromosome, which can be used for restoring prototrophy as well as for testing the metabolic capacity of budding yeast, by compensating for the possible combinations of *his3*, *leu2*, ura *3*, *met17* (or *lys2)* deficiencies. For their use in *S. cerevisiae*, the plasmids contain a centromeric origin for single copy expression and express the marker genes under native *S. cerevisiae* promoter sequences ^39^. To exploit multiple markers to reduce the plasmid segregation problem for protein expression experiments, we further introduced the uniform multiple cloning site of the pRS300 vector series. For cloning and manipulation in *E. coli*, the shuttle vectors contain a bacterial high-copy replication origin (pUC) and an ampicillin antibiotic resistance marker. Finally, the pHLUM series of the plasmid contains an N-terminal fragment (α-peptide) of the *E. coli* beta galactosidase ( *lacZ*), for blue-white selection in appropriate cloning strains ^40^, and an F1 origin for use in phage libraries. These plasmids can be used for complementing unused auxotrophies in laboratory yeast stains, to express proteins exploiting multiple parallel selection markers, and to study metabolite exchange interactions in synthetic yeast communities.

## Materials and methods

### Strains, media and cultivation conditions

*Escherichia coli* strain *DH5*α was used as plasmid host and strains containing the recombinant plasmids were selected on LB medium with ampicillin (100 µg/ml) and grown at 37°C. Two commonly used *S. cerevisiae* strains in the S288c background, BY4741 ( *MAT*a *his3Δ1 leu2Δ0 met17Δ0 ura3Δ0*) and BY4742 ( *MAT*α *his3Δ1 leu2Δ0 lys2Δ0 ura3Δ0*) ^4^, were used to test for genetic complementation of auxotrophic requirements by the plasmids created. The strains were grown in YPD (2% Glucose, 20 g/l peptone (Bacto™), 10 g/l yeast extract (Bacto™)) or synthetic minimal (SM) medium (2% glucose, 6.8 g/l yeast nitrogen base), as indicated. To enable growth of auxotrophic strains, the SM medium was supplemented with 20 mg/l histidine, 60 mg/l leucine, 20 mg/l uracil, 20 mg/l methionine and/or 50 mg/l lysine as indicated.

### Plasmid construction

For site directed mutagenesis the Quikchange Lightning kit (Agilent) was used according to manufacturer guidelines, taking 50 ng of plasmid DNA as a template and 6.3 µM of each oligonucleotide (primers O09-O12, Table 1) to a total volume of 25 µl. The manufacturer's recommended cycling parameters, with a 2.5 min extension time, were followed.

**Table 1.** List of oligonucleotides used to create pHLUM, pHLUM (version 2) and pHLUK.

Primer ID	Sequence (5′-to-3′)	Application
O01	GACGGATCCTCGACTACGTCGTAAGGCCGT	amplification of *LEU2* from pRS425, *BamHI* restriction site in leader sequence
O02	TCACTCGAGGGCGCGCCATCGAGGAGAACTTCTAGTA	amplification of *LEU2* from pRS425, *AscI* and *XhoI* restriction sites in leader sequence
O03	ATGGCGCGCCTGATGCGGTATTTTCTCCTT	amplification of *URA3* from p426GPD, *AscI* restriction site in leader sequence
O04	GGCCTCGAGGCATGCGATTCGGTAATCTCCGAACA	amplification of *URA3* from p426GPD, *SphI* and *XhoI* restriction sites in leader sequence
O05	ATCGCATGCGCCATCCTCATGAAAACTGT	amplification of *MET17* from pRS411, *SphI* restriction site in leader sequence
O06	CATCTCGAGCTTGTGAGAGAAAGTAGGTT	amplification of *MET17* from pRS411, *XhoI* restriction site in leader sequence
O07	TAGCGTCGACGCGTCGAGGAAAACTCTTCAATAG	amplification of *LYS2* from BY4741, *SalI* and *MluI* restriction sites in leader sequence
O08	GCTAGCATGCACATATCATACGTAATGCTC	amplification of *LYS2* from BY4741, *SphI* restriction site in leader sequence
O09	CTCTTGAACTCGAGGATCCTATGCGGTGTG	modification by site directed mutagenesis, new *XhoI* and *BamHI* restriction sites
O10	GGTGTTGGCGGACGTCGGGGCTGGCTTAAC	modification by site directed mutagenesis, new *AatII* restriction site
O11	CTAGAACTAGTGGGTCCCCCGGGCTG	modification by site directed mutagenesis, loss of *BamHI* restriction site
O12	CGATACCGTCGACCTGGAGGGGGGGCC	modification by site directed mutagenesis, loss of *XhoI* restriction site
O13	GATCTGTTTAAACTTAATTAACCTAGGA	modification by annealed primer cloning, loss of *BamHI* and addition of a *PmeI* restriction site
O14	GATCTCCTAGGTTAATTAAGTTTAAACA	modification by annealed primer cloning, loss of *BamHI* and addition of a *PmeI* restriction site
O15	TCAGAGCAGATTGTACTGAGAGTGCACCATAATTCAAAATGACACCGATTATTTAAAGCTGCAGCATACGATAT	modification by homologous recombination, excision of *HIS3*
O16	ATATCGTATGCTGCAGCTTTAAATAATCGGTGTCATTTTGAATTATGGTGCACTCTCAGTACAATCTGCTCTGA	modification by homologous recombination, excision of *HIS3*

All other enzymes for molecular cloning were purchased from New England Biolabs (NEB) and used as instructed. Genomic DNA was extracted from yeast with repeated freeze-thawing of cells in a lysis buffer as per previous publication ^36^. DNA from genomic and plasmid templates was amplified with Phusion High-Fidelity DNA Polymerase (Finnzymes) supplemented with the CES combinatorial enhancer solution to increase primer specificity as described previously ^37^.

Plasmids were isolated both from *E. coli* and *S. cerevisiae* with the QIAprep Spin Miniprep Kit (Qiagen). For the latter, a Qiagen protocol (Michael Jones, Chugai Institute for Molecular Medicine, Ibaraki, Japan, https://www.qiagen.com/gb/resources/resourcedetail?id=5b59b6b3-f11d-4215-b3f7-995a95875fc0&lang=en) was used. The protocol employs 425–600 μm acid-washed glass beads (Sigma) for mechanical lysis (30 sec, 6.5 M/s in a FastPrep®-24 Instrument (MP Biomedicals)). For homologous recombination to construct pLUK, two complementary primers were designed with 5’ and 3’ leader sequences homologous to plasmid 5’ and 3’ of the *HIS3* marker gene (primers O15-O16, Table 1). The oligonucleotides were annealed by heating to 95°C then gradual cooling to RT. The plasmid pHLUK was linearized by cutting inside the *HIS3* sequence with *Msc*I. The yeast strain BY4741 was transformed with 100 ng of cut vector and 500 ng of annealed primers and was selected on SM medium supplemented with histidine. Clones with successful homologous recombination events were identified by failure to grow on SM medium without histidine.

### Yeast transformation

Yeast strains were transformed with a high efficiency lithium acetate, PEG, salmon sperm protocol using 300 ng of plasmid per reaction ^38^.

## Results

### Generation of the plasmid backbone for pHLUM (version 2) series, to complement auxotrophies in BY4741 and isogenic strains

For the genetic complementation of the commonly used auxotrophic lesions in *HIS3*, *LEU2*, *URA3* and *MET17* we have previously constructed the pHLUM minichromome (Addgene ID 40276) ^33^. The plasmid is based on pRS313 ^39^, contains a centromer, and an autonomous replication sequence, and the *HIS3* marker as derived from the pRS313 backbone. The additional marker genes *LEU2*, *URA3* and *MET17* were cloned from other popular yeast plasmids (pRS425 ^39^, p426GPD ^41^ and pRS411 ^4^ and were placed between unique restriction sites, so that they can be individually excised ^33^, primers O01 - O06, Table 1). The three-gene insert is flanked by *Bam*HI and *Xho*I and unique restriction sites *Asc*I and *Sph*I were designed between *LEU2*/ *URA3* and *URA3*/ *MET17,* respectively, to allow for selective excision of the individual markers. However, in this original version of pHLUM, the *HIS3* marker cannot be removed in a straightforward manner.

**Figure 1.**
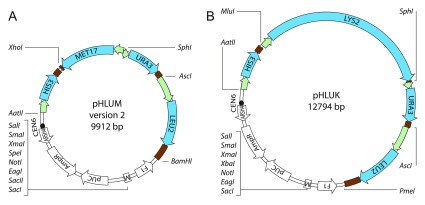
pHLUM (version 2) and pHLUK. Physical maps of pHLUM (version 2) and pHLUK minichromosomes, the centromeric parents for the generated *S. cerevisiae* vector series. pHLUM (version 2) expresses *HIS3, LEU2, URA3 and MET17* to complement auxotrophies in BY4741/ *MAT*a strains of the knock-out collection, while pHLUK expresses *HIS3, LEU2, URA3 and LYS2, for the BY4742/MATα* series.

In order to improve the usefulness of the minichromosome, we decided to redesign the plasmid backbone, replacing all 4 marker genes but leaving the multiple cloning site of pRS313 intact. With a site directed mutagenesis strategy, we added an *Aat*II restriction site 5’ and *Xho*I and *Bam*HI sites 3’ of *HIS3* (primers O09-O10, Table 1). In the same reaction we eliminated the *Bam*HI and *Xho*I recognition sites from the multiple cloning site by exchanging two bases and preserving the sequence of the *lacZ* ɑ-peptide (primers O11-O12, Table 1). With the new restriction sites available and the absence of *Xho*I and *Bam*HI in the multiple cloning site, the DNA fragment containing *LEU2*, *URA3* and *MET17* could be excised from pHLUM with *Xho*I and *Bam*HI and integrated 3’ of the *HIS3* gene on the modified pRS313 vector. The resulting plasmid was named pHLUM (plasmid *HIS3 LEU2 URA3 MET15*) (version 2). It maintains 8 unique endonuclease recognition sites in the multiple cloning site and the capacity for colorimetric *lacZ* complementation assays ( Figure 1A).

### Generation of the plasmid backbone for pHLUK series, to complement auxotrophies in BY4742 isogenic strains

In the typical *MAT*α derivatives of the *S. cerevisiae* gene deletion collection (i.e. BY4742), *LYS2* is deleted while the *MET17* marker is wild-type. We used pHLUM (version 2) as a template and exchanged the *MET17* marker with *LYS2* to create an analoguos vector series (pHLUK). The *LYS2* coding sequence contains recognition sites for both *Xho*I and *Bam*HI. We therefore removed the *Bam*HI site from pHLUM (version 2), and introduced at the same position a recognition sequence for *Pme*I. To this end we synthesised two complementary oligonucleotides (O13-O14, Table 1) and annealed them by heating to 95°C and gradual cooling to RT to yield a small double stranded DNA segment containing a *PmeI* site and cohesive ends to the *Bam*HI digested pHLUM (version 2). The digested vector was dephosphorylated with Antarctic phosphatase (NEB), the annealed primers phosphorylated with polynucleotide kinase (NEB) and then ligated by T4 DNA ligase abolishing the recognition site for *Bam*HI. Next, we amplified the *LYS2* gene from BY4741 genomic DNA (O07-O08, Table 1) including the promoter and terminator regions according to the yeast promoter atlas ^42^. Primer O07 contained recognition sites for *Sal*I and *Mlu*I and primer O08 for *Sph*I ( Table 1). The modified plasmid was digested with *Xho*I and *Sph*I and the *MET17* marker replaced with the *LYS2* PCR product digested with *Sal*I/ *Sph*I. The cohesive ends of *Sal*I and *Xho*I DNA fragments are compatible and abolish their recognition sites upon ligation. The *Mlu*I site allows digestion of the vector between *LYS2* and *HIS3* and excision of either marker ( Figure 1B).

### Generation of pHLUM and pHLUK derivatives possessing all marker combinations

**Figure 2.**
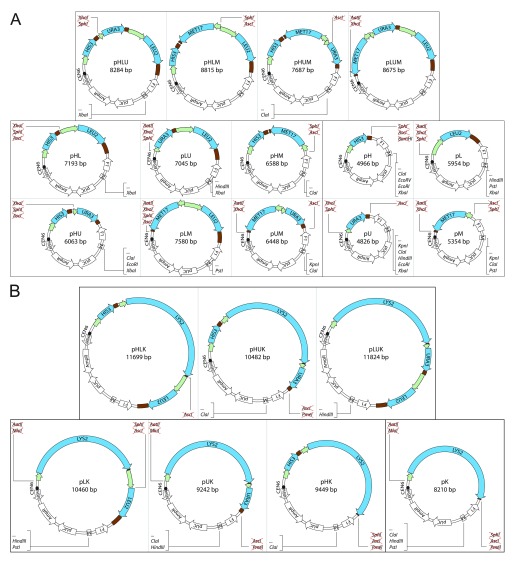
A plasmid series to restore prototrophy in derivatives of BY4741 and BY4742. ( **A**) The plasmids are generated from pHLUM (version 2) containing *HIS3*, *LEU2*, *URA3* and *MET17*, and ( **B**) pHLUK containing *HIS3*, *LEU2*, *URA3* and *LYS2* expressed under control of *S. cerevisiae* promoter and terminator sequences. Unique restriction sites between the marker genes and in the multiple cloning site (M) are indicated in the parent pHLUM (version 2) and pHLUK ( Figure 1). Loss or acquisition of unique restriction sites is highlighted in the individual vector maps.

The unique endonuclease recognition sites between each of the marker genes facilitated the creation of the 21 derivatives of pHLUM (version 2) and pHLUK containing between 1 and 3 marker genes, in all possible combinations. The marker genes were excised by digestion with appropriate endonucleases, the 3' and 5’ overhangs were removed or filled in with T4 DNA polymerase and the plasmid ligated with T4 ligase ( Table 2, Figure 2). The plasmid pLUK was generated using homologous recombination in yeast.

**Table 2.** A plasmid series to complement auxotrophic markers *HIS3, LEU2, URA3, MET17* or *LYS2*, in 23 possible combinations.

Plasmid	Addgene ID	Marker genes	Parental plasmid	Cloning strategy
pHLUM (version 2)	64166	*HIS3 LEU2 URA3 MET17*	pRS313 and pHLUM (ID 40276)	SD, RE
pHLU	64167	*HIS3 LEU2 URA3*	pHLUM (version 2)	RE, BE
pHLM	64168	*HIS3 LEU2 MET17*	pHLUM (version 2)	RE, BE
pLUM	64169	*LEU2 URA3 MET17*	pHLUM (version 2)	RE, BE
pHUM	64170	*HIS3 URA3 MET17*	pHLUM (version 2)	RE, BE
pHL	64171	*HIS3 LEU2*	pHLUM (version 2)	RE, BE
pHU	64172	*HIS3 URA3*	pHUM	RE, BE
pHM	64173	*HIS3 MET17*	pHLUM (version 2)	RE, BE
pLU	64174	*LEU2 URA3*	pHLUM (version 2)	RE, BE
pLM	64175	*LEU2 MET17*	pLUM	RE, BE
pUM	64176	*URA3 MET17*	pHUM	RE, BE
pL	64177	*LEU2*	pHLUM (version 2)	RE, BE
pH	64178	*HIS3*	pHLUM (version 2)	RE, BE
pM	64179	*MET17*	pLUM	RE, BE
pU	64180	*URA3*	pLU	RE, BE
pHLUK	64181	*HIS3 LEU2 LYS2 URA3*	pHLUM (version 2)	AP, RE, BE
pHLK	64182	*HIS3 LEU2 LYS2*	pHLUK	RE, BE
pHUK	64183	*HIS3 URA3 LYS2*	pHLUK	RE, BE
pLUK	64184	*LEU2 URA3 LYS2*	pHLUK	HR
pUK	64185	*URA3 LYS2*	pHUK	RE, BE
pLK	64186	*LEU2 LYS2*	pHLK	RE, BE
pHK	64187	*HIS3 LYS2*	pHLUK	RE, BE
pK	64188	*LYS2*	pHK	RE, BE

SD, site directed mutagenesis; RE, restriction endonuclease; BE, blunt end ligation; HR, homologous recombination; AP, annealed primer cloning

**Figure 3.**
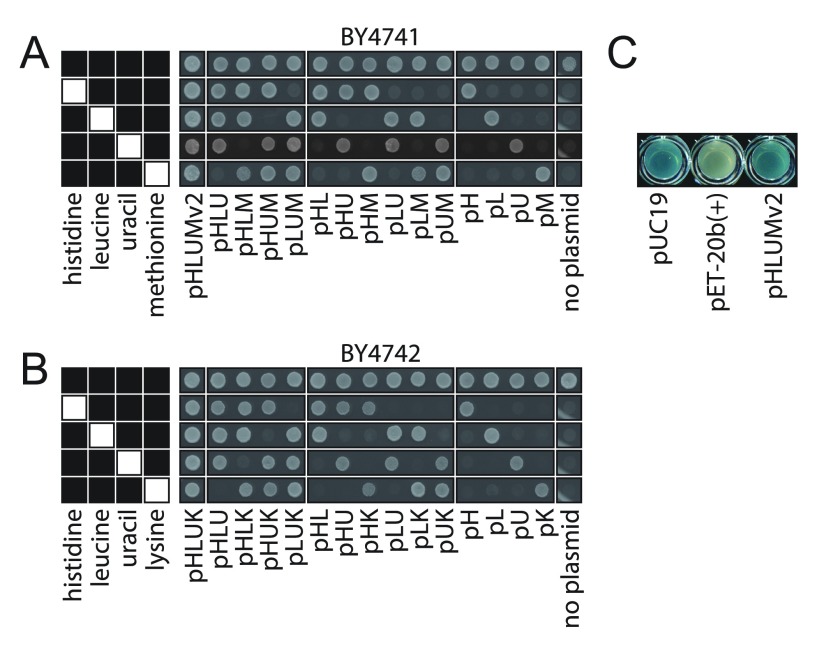
Transformation of BY4741 and BY4742 with the 23 plasmids restores growth on selective media ( **A**) *S. cerevisiae* haploid strains, BY4741 ( *MAT*a *his3Δ1 leu2Δ0 met17Δ0 ura3Δ0*) and ( **B**) BY4742 ( *MAT*α *his3Δ1 leu2Δ0 lys2Δ0 ura3Δ0*) ^4^ were transformed with each of the 23 centromeric plasmids from the pHLUM (version 2) and pHLUK series and spotted onto synthetic minimal medium containing four or all five supplements of 20 mg/l histidine, 60 mg/l leucine, 20 mg/l uracil, 20 mg/l methionine or 50 mg/l lysine, as indicated in black in left hand key. ( **C**) Use of the plasmid series for colorimetric *lacZ* assays: *DH5*α transformed with the *lacZ* containing plasmid pUC19, the *lacZ* missing plasmid pET-20b(+) and pHLUM (version 2) (pHLUMv2), parent of 23 plasmid series. A shift from white to blue on X-Gal containing LB medium due to the presence of a partial *lacZ* sequence.

The completed plasmids were re-sequenced, which led to some corrections compared to the Genebank deposited version of pRS313 (GenBank: U03439.1) ( Supplementary material). Successful genetic complementation of auxotrophic markers is illustrated upon transforming BY4741 and BY4742 strains, with the generated plasmids, and subsequent scoring of their growth on selective media for histidine, leucine, uracil and methionine or lysine, respectively. The plasmids restored all auxotrophies in BY4741 and BY4742 in the desired combination ( Figure 3). Further, we tested the functionality of the *lacZ* α-peptide sequence retained in the pHLUM (version 2) series for blue-white selection, by transforming them in *DH5*α ( Figure 3C). On X-Gal containing medium, a blue colour shift was observed.

## Discussion

Due to the physiological impact of auxotrophy one would in an ideal world conduct all yeast experiments in prototrophic backgrounds and, if the objective of the experiment is to study a physiological process, use cells grown in minimal nutrient medium. However, most existing *Saccharomyces* lab strain resources are auxotrophic, and a majority of genetic techniques depend on the ability to select with genetic markers. The switch to antibiotic resistance markers is not a viable alternative to auxotrophies in many cases, as antibiotics can be expensive, are prone to persistence of sensitive cells, and by interfering with translation or transcription, have strong biological effects on their own ^43, 44^. We have noticed in our previous work, that a useful workaround, or compromise, for many applications is to complement the unused auxotrophic marker mutation with a multi-gene containing, single copy, centromeric single copy plasmid (minichromosome) that compensates for metabolic deficiencies present in the cell ^28, 33^. By nature, introducing an episome adds a new constraint due to its segregation. However, we found that the four metabolic genes on the pHLUM minichromosome provide selective advantage also under nutrient-rich growth conditions, so that cells retain the vector even in the absence of selection pressure ^33^. Further, we tested for copy number effects, and found that the expression of *HIS3, LEU2, MET15* and *URA3* from the minichromosome fully suffices the biosynthetic needs ^33^. A situation in which all cells are provided with a high concentration of nutrients, as would occur with media supplementation, may also be less native to cells community where, usually, a certain fraction of cells are dependent on metabolite exchange ^25, 45^. For the typical experiment, the constraints arising from segregation of a single-copy minichromosome that restores auxotrophy, are hence much smaller compared to the problems caused by the use of nutrient supplemented media and auxotrophic strain backgrounds.

To support the work with prototrophic yeasts, we present here 23 minichromosomal vectors for restoring prototrophy in popular laboratory strains of budding yeast. These plasmids compensate for histidine ( *HIS3*), leucine ( *LEU2*), uracil ( *URA3*), methionine ( *MET17*) and lysine ( *LYS2*) deficiencies and combinations thereof, which have been introduced into many yeast strains derived from the S288c background. Furthermore, the multiple cloning array is compatible with the widely used pRS300 plasmid series and provides unique restriction sites to facilitate cloning of genes of interest. The different marker genes of these vectors also enable expression analysis in various genetic backgrounds. The main intended application of these plasmids is to restore auxotrophy in laboratory strains and to be able to conduct experiments in minimal nutrient supplemented medium. In this way, the effect of amino acid and nucleotide biosynthetic metabolism, which is responsible for a major fraction of the metabolic flux of a cell, as well as has a profound impact on gene expression and physiology ^28^, can be studied.

Another application for these plasmids is to study metabolic cooperation in self establishing yeast communities (SeMeCo). It has been known for a long time that a subpopulation of plasmid free cells can co-grow alongside plasmid containing cells, despite using nutrient selection ^2, 46– 48^. In our lab we have exploited this property to study metabolite exchange interactions between cells, and developed a system of self-establishing metabolically cooperating communities (SeMeCo) in which a series of auxotrophs cooperate to enable the growth of a yeast community ^25, 45^. This system exploits plasmid segregation, starting from an initially self-supporting cell, that grows progressively into an increasingly heterogeneous population, which is able to proliferate on the basis of nutrient exchange occuring between yeast cells. The progressive self-establishment overcomes a failure that is typically observed when yeast auxotrophs are forced to establish a bilateral cooperation. Other than through self-establishment, this lethality is overcome by yeast cells being genetically modified to artificially overproduce metabolites needing to be exchanged. The synthetic communities generated in this way have been intensively studied and serve as a model for ecological metabolite exchange interactions ^49– 52^. The new vector series, having multiple auxotrophic markers on single centromeric plasmids, can support the design of such communities, as it removes the likelihood of recombination occurring when multiple plasmids are used in parallel, to obtain the desired auxotrophic background.

Finally, the uniform multiple cloning site (MCS) in the plasmid series allows for the inclusion of marker proteins, such as GFP or beta-galactosidase, to track individual cell types in SeMeCo communities, that reveal phenotypic heterogeneity at the single cell level ^45^. This MCS further allows the use of these plasmids for the recombinant expression of proteins. Here, one can profit from multiple auxotrophic markers on one plasmid to improve selection and reduce plasmid segregation rate, so that (as long as no disadvantageous protein is expressed) the plasmids can be maintained in rich medium in the absence of selection pressure ^33^. This strategy of using multiple markers in parallel can further be exploited to increase selection pressure, to counteract the well-known issue of clonal selection phenotypes, emerging when overexpressing recombinant proteins.

In summary, to test both the effects of prototrophy as well as the metabolic capacity of budding yeast, to design self-establishing metabolically cooperating communities, and to profit from multiple selection markers when expressing proteins, we present a series of centromeric plasmids that can compensate for histidine ( *HIS3*), leucine ( *LEU2*), uracil ( *URA3*), methionine ( *MET17*) or lysine ( *LYS2*) deficiencies in 23 possible combinations. These vectors are accessible individually ( Table 2) or as a Kit from Addgene ( www.addgene.org/kits/prototrophy/). We hope they benefit the community when analysing the importance of biosynthetic metabolism for gene function, gene expression, physiology and metabolite exchange.

## Data availability

The data referenced by this article are under copyright with the following copyright statement: Copyright: © 2016 Mülleder M et al.

The full sequences of the 23 plasmids are deposited in Addgene under the ID numbers listed in Table 1.

## Supplementary material

**Plasmid sequences used in Figure 1 and Figure 2.** Plasmid sequences for the 23 vectors have been assembled from the pHLUM and pRS300 vector sequences, respectively, and verified and corrected upon resequencing.

## References

[1] Tong AH Evangelista M Parsons AB: Systematic genetic analysis with ordered arrays of yeast deletion mutants. *Science.* 2001;294(5550):2364–8. 10.1126/science.1065810 11743205

[2] Pronk JT: Auxotrophic yeast strains in fundamental and applied research. *Appl Environ Microbiol.* 2002;68(5):2095–100. 10.1128/AEM.68.5.2095-2100.2002 11976076 PMC127579

[3] Rothstein RJ: One-step gene disruption in yeast. *Methods Enzymol.* 1983;101:202–11. 10.1016/0076-6879(83)01015-0 6310324

[4] Brachmann CB Davies A Cost GJ: Designer deletion strains derived from *Saccharomyces cerevisiae* S288C: a useful set of strains and plasmids for PCR-mediated gene disruption and other applications. *Yeast.* 1998;14(2):115–32. 10.1002/(SICI)1097-0061(19980130)14:2<115::AID-YEA204>3.0.CO;2-2 9483801

[5] Winzeler EA Shoemaker DD Astromoff A: Functional characterization of the *S. cerevisiae* genome by gene deletion and parallel analysis. *Science.* 1999;285(5429):901–6. 10.1126/science.285.5429.901 10436161

[6] Fink GR: GENE-ENZYME RELATIONS IN HISTIDINE BIOSYNTHESIS IN YEAST. *Science.* 1964;146(3643):525–7. 10.1126/science.146.3643.525 14190241

[7] Lacroute F: Regulation of pyrimidine biosynthesis in *Saccharomyces cerevisiae*. *J Bacteriol.* 1968;95(3):824–32. 5651325 10.1128/jb.95.3.824-832.1968 PMC252099

[8] Masselot M De Robichon-Szulmajster H: Methionine biosynthesis in *Saccharomyces cerevisiae*. I. Genetical analysis of auxotrophic mutants. *Mol Gen Genet.* 1975;139(2):121–32. 10.1007/BF00264692 1101032

[9] Satyanarayana T Umbarger HE Lindegren G: Biosynthesis of branched-chain amino acids in yeast: regulation of leucine biosynthesis in prototrophic and leucine auxotrophic strains. *J Bacteriol.* 1968;96(6):2018–24. 5724970 10.1128/jb.96.6.2018-2024.1968 PMC252553

[10] Giaever G Chu AM Ni L: Functional profiling of the *Saccharomyces cerevisiae* genome. *Nature.* 2002;418(6896):387–91. 10.1038/nature00935 12140549

[11] Ryan O Shapiro RS Kurat CF: Global gene deletion analysis exploring yeast filamentous growth. *Science.* 2012;337(6100):1353–6. 10.1126/science.1224339 22984072

[12] Huh W-K, Falvo JV Gerke LC: Global analysis of protein localization in budding yeast. *Nature.* 2003;425(6959):686–91. 10.1038/nature02026 14562095

[13] Sopko R Huang D Preston N: Mapping pathways and phenotypes by systematic gene overexpression. *Mol Cell.* 2006;21(3):319–30. 10.1016/j.molcel.2005.12.011 16455487

[14] Ghaemmaghami S Huh W-K, Bower K: Global analysis of protein expression in yeast. *Nature.* 2003;425(6959):737–41. 10.1038/nature02046 14562106

[15] Ross-Macdonald P Coelho PS Roemer T: Large-scale analysis of the yeast genome by transposon tagging and gene disruption. *Nature.* 1999;402(6760):413–8. 10.1038/46558 10586881

[16] Breslow DK Cameron DM Collins SR: A comprehensive strategy enabling high-resolution functional analysis of the yeast genome. *Nat Methods.* 2008;5(8):711–8. 10.1038/nmeth.1234 18622397 PMC2756093

[17] Mnaimneh S Davierwala AP Haynes J: Exploration of essential gene functions via titratable promoter alleles. *Cell.* 2004;118(1):31–44. 10.1016/j.cell.2004.06.013 15242642

[18] Kofoed M Milbury KL Chiang JH: An Updated Collection of Sequence Barcoded Temperature-Sensitive Alleles of Yeast Essential Genes. *G3 (Bethesda).* 2015;5(9):1879–87. 10.1534/g3.115.019174 26175450 PMC4555224

[19] Ben-Aroya S Coombes C Kwok T: Toward a comprehensive temperature-sensitive mutant repository of the essential genes of *Saccharomyces cerevisiae*. *Mol Cell.* 2008;30(2):248–58. 10.1016/j.molcel.2008.02.021 18439903 PMC4130347

[20] Decottignies A Sanchez-Perez I Nurse P: *Schizosaccharomyces pombe* essential genes: a pilot study. *Genome Res.* 2003;13(3):399–406. 10.1101/gr.636103 12618370 PMC430286

[21] Kim DU Hayles J Kim D: Analysis of a genome-wide set of gene deletions in the fission yeast *Schizosaccharomyces pombe*. *Nat Biotechnol.* 2010;28(6):617–23. 10.1038/nbt.1628 20473289 PMC3962850

[22] Schwarzmüller T Ma B Hiller E: Systematic phenotyping of a large-scale *Candida glabrata* deletion collection reveals novel antifungal tolerance genes. *PLoS Pathog.* 2014;10(6):e1004211. 10.1371/journal.ppat.1004211 24945925 PMC4063973

[23] Noble SM French S Kohn LA: Systematic screens of a *Candida albicans* homozygous deletion library decouple morphogenetic switching and pathogenicity. *Nat Genet.* 2010;42(7):590–8. 10.1038/ng.605 20543849 PMC2893244

[24] Colot HV Park G Turner GE: A high-throughput gene knockout procedure for *Neurospora* reveals functions for multiple transcription factors. *Proc Natl Acad Sci USA.* 2006;103(27):10352–7. 10.1073/pnas.0601456103 16801547 PMC1482798

[25] Campbell K Vowinckel J Mülleder M: Self-establishing communities enable cooperative metabolite exchange in a eukaryote. *eLife.* 2015;4: pii: e09943. 10.7554/eLife.09943 26499891 PMC4695387

[26] Nissen TL Schulze U Nielsen J: Flux distributions in anaerobic, glucose-limited continuous cultures of *Saccharomyces cerevisiae*. *Microbiology.* 1997;143(Pt 1):203–18. 10.1099/00221287-143-1-203 9025295

[27] Park JO Rubin SA Xu YF: Metabolite concentrations, fluxes and free energies imply efficient enzyme usage. *Nat Chem Biol.* 2016;12(7):482–9. 10.1038/nchembio.2077 27159581 PMC4912430

[28] Alam MT Zelezniak A Mülleder M: The metabolic background is a global player in *Saccharomyces* gene expression epistasis. *Nat Microbiol.* 2016;1: 15030. 10.1038/nmicrobiol.2015.30 27572163 PMC5131842

[29] Petek Çakar Z Sauer U Bailey JE: Metabolic engineering of yeast: the perils of auxotrophic hosts. *Biotechnol Lett.* 1999;21(7):611–6. 10.1023/A:1005576004215

[30] Canelas AB Harrison N Fazio A: Integrated multilaboratory systems biology reveals differences in protein metabolism between two reference yeast strains. *Nat Commun.* 2010;1: 145. 10.1038/ncomms1150 21266995

[31] Gupta JC Mukherjee KJ: Stability studies of recombinant *Saccharomyces cerevisiae* in the presence of varying selection pressure. *Biotechnol Bioeng.* 2002;78(5):475–88. 10.1002/bit.10213 12115116

[32] Kokina A Kibilds J Liepins J: Adenine auxotrophy--be aware: some effects of adenine auxotrophy in *Saccharomyces cerevisiae* strain W303-1A. *FEMS Yeast Res.* 2014;14(5):697–707. 10.1111/1567-1364.12154 24661329

[33] Mülleder M Capuano F Pir P: A prototrophic deletion mutant collection for yeast metabolomics and systems biology. *Nat Biotechnol.* 2012;30(12):1176–8. 10.1038/nbt.2442 23222782 PMC3520112

[34] Niederberger P Miozzari G Hütter R: Biological role of the general control of amino acid biosynthesis in *Saccharomyces cerevisiae*. *Mol Cell Biol.* 1981;1(7):584–93. 10.1128/MCB.1.7.584 9279372 PMC369706

[35] Wagner A Fell DA: The small world inside large metabolic networks. *Proc Biol Sci.* 2001;268(1478):1803–10. 10.1098/rspb.2001.1711 11522199 PMC1088812

[36] Harju S Fedosyuk H Peterson KR: Rapid isolation of yeast genomic DNA: Bust n’ Grab. *BMC Biotechnol.* 2004;4(1):8. 10.1186/1472-6750-4-8 15102338 PMC406510

[37] Ralser M Querfurth R Warnatz HJ: An efficient and economic enhancer mix for PCR. *Biochem Biophys Res Commun.* 2006;347(3):747–51. 10.1016/j.bbrc.2006.06.151 16842759

[38] Gietz RD Schiestl RH: High-efficiency yeast transformation using the LiAc/SS carrier DNA/PEG method. *Nat Protoc.* 2007;2(1):31–4. 10.1038/nprot.2007.13 17401334

[39] Sikorski RS Hieter P: A system of shuttle vectors and yeast host strains designed for efficient manipulation of DNA in *Saccharomyces cerevisiae*. *Genetics.* 1989;122(1):19–27. 2659436 10.1093/genetics/122.1.19 PMC1203683

[40] Cronan JE Jr Narasimhan ML Rawlings M: Insertional restoration of beta-galactosidase alpha-complementation (white-to-blue colony screening) facilitates assembly of synthetic genes. *Gene.* 1988;70(1):161–70. 10.1016/0378-1119(88)90114-X 2853687

[41] Mumberg D Müller R Funk M: Yeast vectors for the controlled expression of heterologous proteins in different genetic backgrounds. *Gene.* 1995;156(1):119–22. 10.1016/0378-1119(95)00037-7 7737504

[42] Chang DT Huang CY Wu CY: YPA: an integrated repository of promoter features in *Saccharomyces cerevisiae*. *Nucleic Acids Res.* 2011;39(Database issue):D647–52. 10.1093/nar/gkq1086 21045055 PMC3013683

[43] Andersson DI Levin BR: The biological cost of antibiotic resistance. *Curr Opin Microbiol.* 1999;2(5):489–93. 10.1016/S1369-5274(99)00005-3 10508723

[44] Andersson DI: The biological cost of mutational antibiotic resistance: any practical conclusions? *Curr Opin Microbiol.* 2006;9(5):461–5. 10.1016/j.mib.2006.07.002 16890008

[45] Campbell K Vowinckel J Ralser M: Cell-to-cell heterogeneity emerges as consequence of metabolic cooperation in a synthetic yeast community. *Biotechnol J.* 2016;.11(9):1169–78. 10.1002/biot.201500301 27312776 PMC5031204

[46] Christianson TW Sikorski RS Dante M: Multifunctional yeast high-copy-number shuttle vectors. *Gene.* 1992;110(1):119–22. 10.1016/0378-1119(92)90454-W 1544568

[47] Meinander NQ Hahn-Hägerdal B: Fed-batch xylitol production with two recombinant *Saccharomyces cerevisiae* strains expressing XYL1 at different levels, using glucose as a cosubstrate: a comparison of production parameters and strain stability. *Biotechnol Bioeng.* 1997;54(4):391–9. 10.1002/(SICI)1097-0290(19970520)54:4<391::AID-BIT12>3.0.CO;2-J 18634106

[48] Sardonini CA Dibiasio D: A model for growth of *Saccharomyces cerevisiae* containing a recombinant plasmid in selective media. *Biotechnol Bioeng.* 1987;29(4):469–75. 10.1002/bit.260290410 18576474

[49] Momeni B Brileya KA Fields MW: Strong inter-population cooperation leads to partner intermixing in microbial communities. *eLife.* 2013;2:e00230. 10.7554/eLife.00230 23359860 PMC3552619

[50] Shou W Ram S Vilar JM: Synthetic cooperation in engineered yeast populations. *Proc Natl Acad Sci USA.* 2007;104(6):1877–82. 10.1073/pnas.0610575104 17267602 PMC1794266

[51] Hoek TA Axelrod K Biancalani T: Resource Availability Modulates the Cooperative and Competitive Nature of a Microbial Cross-Feeding Mutualism. *PLoS Biol.* 2016;14(8):e1002540. 10.1371/journal.pbio.1002540 27557335 PMC4996419

[52] Müller MJ Neugeboren BI Nelson DR: Genetic drift opposes mutualism during spatial population expansion. *Proc Natl Acad Sci USA.* 2014;111(3):1037–42. 10.1073/pnas.1313285111 24395776 PMC3903240

