# Supplementary material for: Saccharomyces cerevisiae single-copy plasmids for auxotrophy compensation, multiple marker selection, and for designing metabolically cooperating communities
Source: F1000Res. 2016 Sep 20;5:2351. [Version 1] doi: 10.12688/f1000research.9606.1 (PMC5081161; doi:10.12688/f1000research.9606.1)
Supplement: Peer review history

# Peer review: Referee response for version 1

**DOI:** 10.5256/f1000research.10348.r16547

**Published:** 2016-10-24

**Authors:** Amy A. Caudy

**Affiliations:** 1 Donnelly Centre and Department of Molecular Genetics, University of Toronto, Toronto, ON, Canada

The Ralser group has thoughtfully designed and created a comprehensive set of plasmids suitable for the complementation of various combinations of the widely used leucine ( *leu2*), uracil ( *ura3*), methionine ( *met17*, formerly known as *met15*), histidine ( *his3*), and lysine ( *lys2*) markers. Auxotrophy can have significant effects on a variety of phenotypes, as shown by this group and others (and as well-described in the introduction). This new collection of plasmids provides a convenient source of auxotrophic markers supplied from their endogenous promoters on a CEN/ARS plasmid, which is near single-copy in most situations.

 Not content to make a simple derivative of the pHLUM plasmid they previously created in order to build a prototrophic version of the yeast deletion collection, the group went to significant efforts to preserve several sites including BamHI and XhoI within the blue/white compatible multiple cloning site of their base vector. These plasmids have been fully sequenced in collaboration with Addgene, revealing a handful of mutations not present in the original (theoretical) sequence of the parent plasmid deposited in Genbank more than a decade ago.

 One minor point could be mentioned for clarity -

 The introduction mentions supplementation of "nucleotides" but it is important to note that yeast cells do not take up (phosphorylated) nucleotides directly. Instead, these are dephosphorylated by extracellular phosphatases to nucleosides and then imported. There are also transporters that can efficiently take in nucleobases such as uridine.

I have read this submission. I believe that I have an appropriate level of expertise to confirm that it is of an acceptable scientific standard.

# Peer review: Referee response for version 1

**DOI:** 10.5256/f1000research.10348.r16449

**Published:** 2016-10-10

**Authors:** Campbell Gourlay

**Affiliations:** 1 School of Biosciences, Kent University, Kent, UK

The paper nicely describes the generation and testing of a useful plasmid set that allows complementation of commonly used auxotrophic markers in the budding yeast system. The paper provides clear and adequate descriptions of their construction and therefore represents a solid piece of work that is ready for indexing as it is currently presented.

I have read this submission. I believe that I have an appropriate level of expertise to confirm that it is of an acceptable scientific standard.
